# Expression of the MexXY Aminoglycoside Efflux Pump and Presence of an Aminoglycoside-Modifying Enzyme in Clinical Pseudomonas aeruginosa Isolates Are Highly Correlated

**DOI:** 10.1128/AAC.01166-20

**Published:** 2020-12-16

**Authors:** Alexander Seupt, Monika Schniederjans, Jürgen Tomasch, Susanne Häussler

**Affiliations:** a Department of Molecular Bacteriology, Helmholtz Centre for Infection Research, Braunschweig, Germany; b Department of Molecular Bacteriology, Twincore, Hannover, Germany; c Department of Clinical Microbiology, Rigshospitalet, Copenhagen, Denmark; d Cluster of Excellence RESIST (EXC 2155), Hannover Medical School, Hannover, Germany

**Keywords:** MexXY, aminoglycoside resistance, aminoglycoside-modifying enzymes, antibiotic efflux, antibiotic resistance

## Abstract

The impact of MexXY efflux pump expression on aminoglycoside resistance in clinical Pseudomonas aeruginosa isolates has been debated. In this study, we found that, in general, elevated *mexXY* gene expression levels in clinical P. aeruginosa isolates confer to slight increases in aminoglycoside MIC levels; however, those levels rarely lead to clinically relevant resistance phenotypes. The main driver of resistance in the clinical isolates studied here was the acquisition of aminoglycoside-modifying enzymes (AMEs). Nevertheless, acquisition of an AME was strongly associated with *mexY* overexpression. In line with this observation, we demonstrate that the introduction of a gentamicin acetyltransferase confers to full gentamicin resistance levels in a P. aeruginosa type strain only if the MexXY efflux pump was active. We discuss that increased *mexXY* activity in clinical AME-harboring P. aeruginosa isolates might affect ion fluxes at the bacterial cell membrane and thus might play a role in the establishment of enhanced fitness that extends beyond aminoglycoside resistance.

## Abstract (precis)

The impact of MexXY efflux pump expression on aminoglycoside resistance in clinical Pseudomonas aeruginosa isolates has been debated. In this study, we found that, in general, elevated *mexXY* gene expression levels in clinical P. aeruginosa isolates confer to slight increases in aminoglycoside MIC levels; however, those levels rarely lead to clinically relevant resistance phenotypes. The main driver of resistance in the clinical isolates studied here was the acquisition of aminoglycoside-modifying enzymes (AMEs).

## INTRODUCTION

The metabolically versatile bacterium Pseudomonas aeruginosa is known to cause a wide range of opportunistic infections and is recognized as one of the leading causes of severe hospital-acquired acute infections (1–6). In cystic fibrosis (CF), P. aeruginosa frequently establishes lifelong chronic infections associated with hyperinflammation, severe tissue damage, and, ultimately, high mortality rates (7–9). Although antimicrobial therapy is a cornerstone of the management of CF, even intensified treatment is often not able to clear the infection (10–12). The recalcitrance of chronic P. aeruginosa infections against antibiotic treatment is only incompletely understood. A known important adaptive resistance trait is the induction of active efflux of antibiotics into the extracellular space (13–15). Multidrug efflux pumps recognize a variety of structurally diverse compounds as their substrates, including different clinically relevant antibiotic classes (3, 16). In P. aeruginosa, there are four efflux pump systems that are of particular interest: MexAB-OprM, MexCD-OprJ, MexEF-OprN, and MexXY-OprM (17, 18). Previous studies have shown that *mexZ*, encoding the negative regulator of MexXY-OprM, is one of the mutational hot spots in CF-derived P. aeruginosa isolates (19–22). P. aeruginosa *mexZ* mutants exhibit constitutive overexpression of MexXY-OprM. This efflux pump was shown to play an important role in the development of resistance against aminoglycosides and fluoroquinolones (23, 24), both being important antibiotic classes in the treatment of CF patients (10).

However, the causal contribution of P. aeruginosa MexXY-OprM pump expression to aminoglycoside resistance in the clinic is disputed. Recently published evolutionary studies did not detect the emergence of *mexXY*-overexpressing strains, even under prolonged aminoglycoside exposure (25, 26). Other studies show only limited aminoglycoside resistance in clinical isolates despite overexpression of the MexXY-OprM efflux pump (27). Nevertheless, the MexXY-OprM-overproducing strains seem to have a fitness advantage when grown under sub-MIC tobramycin concentrations, a condition that especially CF isolates face during chronic lung infections (7).

In this study, we aimed to shed further light on the impact of MexXY efflux pump expression on aminoglycoside resistance in clinical P. aeruginosa isolates. For this purpose, we analyzed the expression of genes encoding the MexXY efflux pump in a recently published collection of 412 clinical P. aeruginosa isolates from a variety of geographical origins and infection sites (28). We found that clinically relevant tobramycin resistance was, in general, not associated with elevated *mexXY* gene expression levels but that the acquisition of aminoglycoside-modifying enzymes (AMEs) was the main driver of resistance. However, acquisition of an AME was strongly associated with *mexY* overexpression, and the full gentamicin acetyltransferase-mediated gentamicin resistance potential was dependent on an active MexXY efflux pump.

## RESULTS

### *mexY* overexpression can be frequently found in clinical P. aeruginosa isolates.

**FIG 1:**
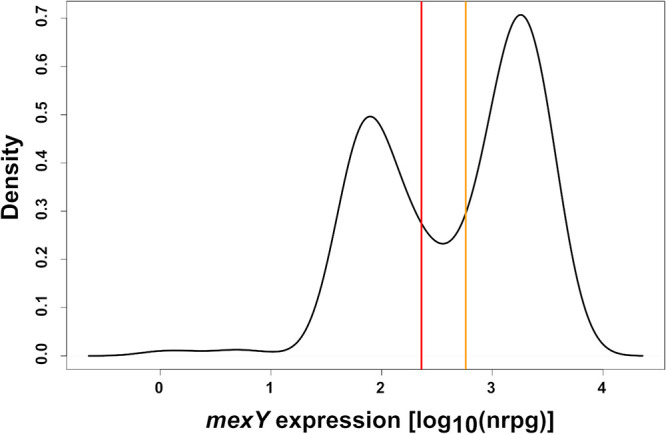
Distribution of *mexY* expression values and cutoff values for high *mexY* expression across clinical P. aeruginosa isolates. The distribution of log_10_ expression values (NRPGs) of *mexY* over all analyzed clinical isolates (*n* = 412) (28) is depicted. Cutoff values for high (orange line) and low (red line) *mexY* expression were defined as the 30% distance from the lowest point between the two maxima of the distribution to the two maxima, respectively.

In this study, we took advantage of previously recorded transcriptome data on 412 clinical P. aeruginosa isolates grown in LB medium until the late logarithmic phase (optical density at 600 nm [OD_600_], 2) (28). Most of the clinical isolates exhibited resistance against at least one class of antibiotics (29), and about one-third of the P. aeruginosa isolates have been isolated from CF patients. We focused on the expression of the MexXY-OprM efflux pump and extracted gene expression values for *mexY* across all isolates. We defined a cutoff value for high *mexY* gene expression by analyzing the distribution of normalized reads per gene (NRPG) over all analyzed clinical isolates (Fig. 1). We found a biphasic pattern of *mexY* gene expression and classified an isolate as overexpressing *mexY* if at least 575.44 NRPG mapped to *mexY* (Fig. 1, orange line). This resulted in the categorization of 53% (*n* = 217) of the clinical isolates as *mexY* overexpressing. Nine percent (*n* = 37) of the isolates exhibited intermediate *mexY* expression values (between 229.09 and 575.44 NRPG) (Fig. 1).

Under noninducing rich medium growth conditions, MexZ acts as a negative regulator of *mexXY* gene expression. We found at least one nonsynonymous mutation in *mexZ* in 86% (*n* = 187) of the *mexY*-overexpressing clinical isolates. There was no clear mutational hot spot within the *mexZ* gene sequence (Fig. S1 in the supplemental material), although isolates with high *mexY* expression levels tend to be enriched in mutations in the N-terminal region of the gene in which the DNA binding domain was identified (22). Nevertheless, our results suggest that inactivation of the negative regulator MexZ leads to constitutive efflux pump overexpression in the majority of our clinical isolates. However, there are additional MexXY-OprM expression regulators which might play a role in our data set, e.g., overexpression of the MexZ antirepressor AmrZ or activating mutations in the sensor histidine kinase of ParRS have been described to positively control *mexXY* expression (22).

### Overexpression of *mexY* correlates with high MexY protein abundance.

**FIG 2:**
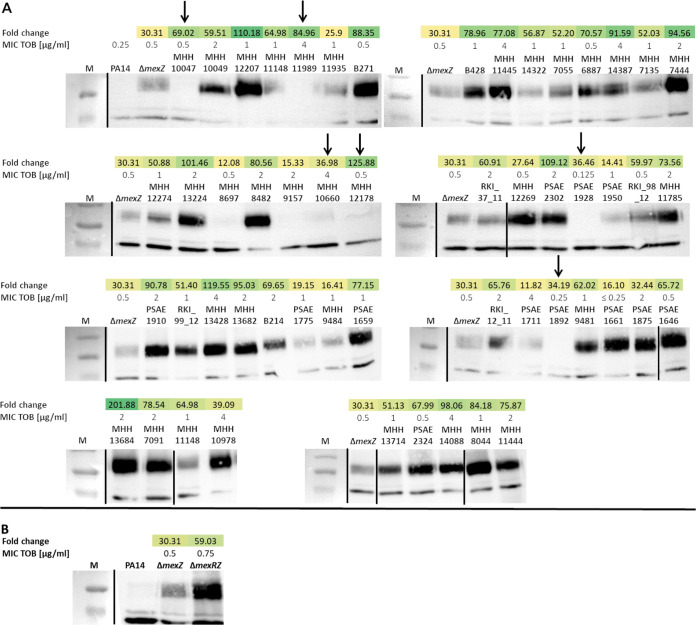
Correlation of MexY protein abundance with *mexY* gene expression level and aminoglycoside resistance. (A) MexY (113-kDa) protein levels were detected by Western blot analysis using a polyclonal anti-MexY antibody in 53 clinical isolates. The results of tobramycin susceptibility testing as reported previously (30) are shown (MIC of tobramycin [MIC TOB] in micrograms per milliliter). *mexY* expression (also reported in reference 30) is indicated as fold-change gene expression compared to the PA14 reference strain. (B) The PA14 Δ*mexZ* or Δ*mexRZ* mutant strains were used as positive controls on each blotting membrane. Arrows indicate strains with *mexY* overexpression on the mRNA level but missing or only faint MexY protein band. M, PageRuler prestained protein ladder. Black bars indicate borders of single Western blotting membranes.

To evaluate whether the constitutively high *mexY* transcript levels are translated into high protein levels, Western blot analysis was performed on a set of 53 clinical P. aeruginosa isolates (30). We included a PA14Δ*mexZ* (31) mutant as a control. Due to the inactivation of the negative regulator, MexZ, PA14Δ*mexZ* is producing elevated levels of the efflux pump (Fig. 2). In six of the clinical isolates, (almost) no MexY protein was detected despite *mexY* overexpression (Fig. 2, arrows). We cannot exclude that variations in the protein sequence impede the detection by the antibody used in the Western blot analysis. Nevertheless, in general, the clinical isolates, which exhibited high *mexY* transcripts, also produced high MexY protein expression levels. For most of the isolates (83%; *n* = 44), these levels were far above the levels of the PA14 Δ*mexZ* mutant. In line with the finding that not all clinical isolates harbored *mexZ* mutations, these results suggest additional levels of MexXY regulation in the clinical isolates studied here. In this respect, it is interesting that in a PA14 strain background with an inactivation of not only *mexZ* but also *mexR* and *mexY* expression was further increased (Fig. 2). MexR represses transcription of the *mexAB-oprM* operon, and OprM is an outer membrane factor that is shared between the MexXY and the MexAB efflux systems. Thus, there seems to be a functional link of the two efflux systems that also impacts *mexY* expression levels.

### Increased *mexY* expression has a limited impact on resistance against tobramycin.

**FIG 3:**
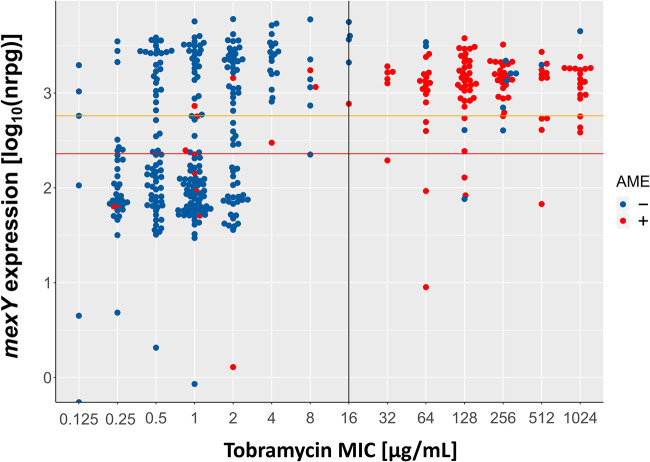
Correlation of tobramycin MIC values with *mexY* gene expression. Expression of *mexY* in 412 clinical P. aeruginosa isolates relative to the tobramycin MIC as reported previously (28, 29). Clinical isolates exhibiting *mexY* expression levels above the orange line were defined to exhibit intermediate, and above the red line exhibit high *mexY* expression levels. The black line delineates the CLSI breakpoint for clinical resistance. The blue color code indicates that the respective clinical isolate does not harbor an aminoglycoside modifying enzyme (AME^−^ isolates), while the red color code indicates that the isolates are AME positive (AME^+^).

**FIG 4:**
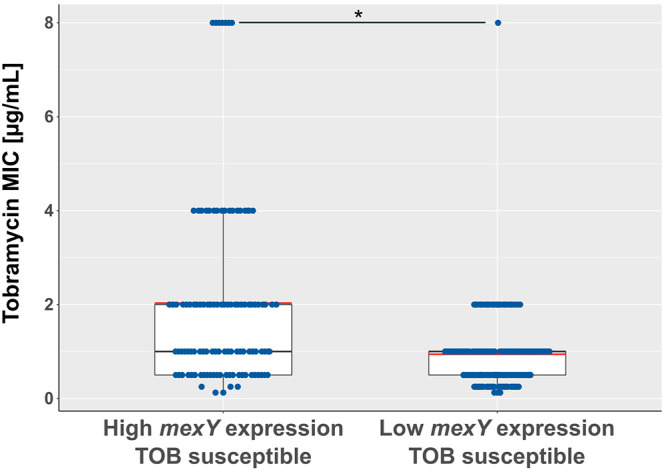
High *mexY* expression correlates with elevated MICs against tobramycin. MICs of tobramycin-susceptible isolates (MIC < 16 μg/ml) were assigned to high and low *mexY* expression levels, respectively. Boxes comprise the MIC values of 50% of the clinical isolates in the respective group. Red line indicates mean. A *P* value of < 0.05 represents statistical significance (Wilcoxon rank sum test).

We next evaluated whether higher expression of *mexXY-oprM* was associated with aminoglycoside resistance. Among the group of *mexY*-overexpressing isolates, 51% (*n* = 110) were classified as tobramycin susceptible according to the standards defined by the Clinical and Laboratory Standards Institute (tobramycin MIC < 16 μg/ml; Fig. 3) (32), indicating that *mexY* expression is not of major importance for exhibiting clinically relevant resistance levels. Nevertheless, we detected a significant yet limited impact of increased *mexY* expression levels on resistance toward tobramycin. Compared to the tobramycin susceptible isolates with low *mexY* expression (*n* = 151), the 110 tobramycin-susceptible isolates with high *mexY* expression exhibited a 2-fold higher mean MIC against tobramycin (2 μg/ml versus 1 μg/ml, respectively; Fig. 4). Our results are in agreement with previously published data demonstrating that efflux pump expression does play—albeit a minor—role in the development of resistance against tobramycin (25, 27).

### The presence of aminoglycoside-modifying enzymes and *fusA1* mutations are drivers of resistance against tobramycin.

Besides efflux pump overexpression, the acquisition of genes encoding for aminoglycoside-modifying enzymes (AME) is well-known to confer to aminoglycoside resistance (33). We therefore screened our clinical isolate collection for the presence of AMEs. Within the group of tobramycin-resistant isolates, 87% (*n* = 111) harbored at least one AME (for AME frequency, see Fig. S2), while in the group of tobramycin-susceptible isolates, only 5% (*n* = 14) contained at least one AME (Fig. 3). The presence of AMEs was thus significantly overrepresented in the resistant isolates (hypergeometric test for overrepresentation; *P* < 0.001), clearly indicating that a main driver for tobramycin resistance is the acquisition of an AME.

Recently, mutations in *fusA* were identified to contribute to elevated resistance levels following prolonged aminoglycoside treatment (20, 26, 34). Thus, we investigated the impact of those mutations on the overall resistance pattern in our data set. Of the overall 48 isolates with nonsynonymous *fusA* mutations, six isolates (12.5%) exhibited clinically relevant (MIC ≥ 16 μg/ml) tobramycin resistance levels that could not be attributed to the presence of an AME. This corresponds to 4.72% of all tobramycin-resistant isolates.

### The full AME potential is dependent on *mexXY* expression.

**FIG 5:**
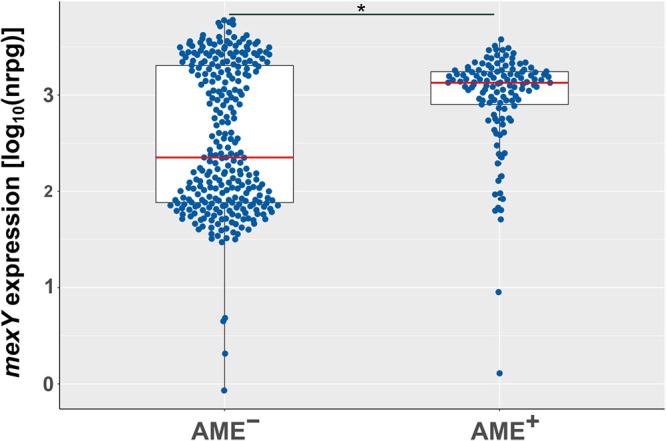
Presence of an AME correlates with high *mexY* expression. Normalized expression values [log_10_(NRPG)] of isolates that were identified as AME containing (AME^+^) and non-AME containing (AME^−^). Boxes comprise 50% of the values; red line indicates median. A *P* value of < 0.01 represents statistical significance (Wilcoxon rank sum test).

Strikingly, 85% (*n* = 94) of the tobramycin-resistant AME-containing (AME^+^) isolates also overexpressed *mexY*. Only few (*n* = 6) tobramycin-resistant isolates that harbored an AME exhibited low *mexY* expression values. Eleven isolates expressed intermediate *mexY* levels (Fig. 3). To test for a possible correlation of the presence of an AME and *mexY* expression, we compared *mexY* expression values of all AME^+^ isolates and all non-AME-containing (AME^−^) isolates. We found a significant 6-fold increased median *mexY* expression in AME^+^ isolates (Fig. 5; *P* < 0.001). Of note, this correlation was specific to *mexY*. No correlation was identified between the presence of an AME and the expression of RND genes encoded in other important efflux systems (*mexB*, *mexD*, and *mexF*) (Fig. S3).

**FIG 6:**
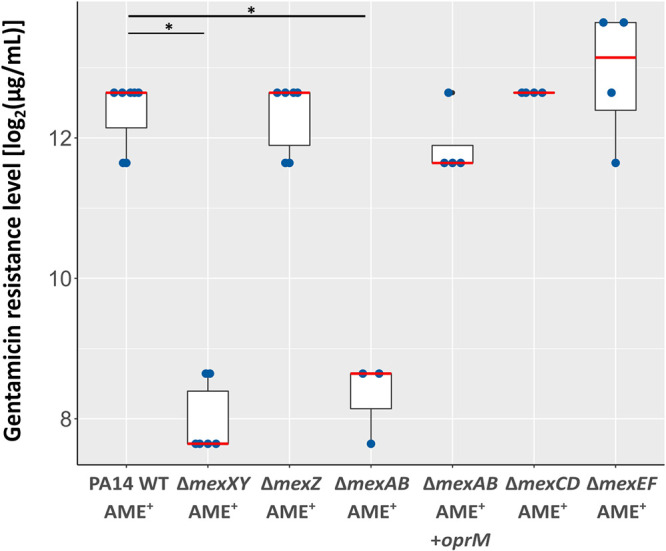
AME-induced gentamicin resistance is impacted by the presence of the MexXY-OprM efflux pump. Gentamicin resistance levels of P. aeruginosa PA14 and mutants thereof. All strains were transformed with the pSEVA621 vector, harboring a gentamicin acetyltransferase (AME^+^) (35). The Δ*mexAB* AME^+^ strain was complemented with pSEVA634::oprM (Δ*mexAB* AME^+^/+*oprM*). pSEVA634 harbors the same acetyltransferase as pSEVA621. Red line depicts the median resistance level of at least 4 biological replicates. A *P* value of < 0.05 represents statistical significance (Wilcoxon rank sum test).

Our results suggest a functional link of *mexXY-oprM* expression and the presence of an acquired AME. To evaluate this further, we constructed clean deletions of structural genes of important “mex” efflux pump systems (PA14Δ*mexAB*, PA14Δ*mexCD*, PA14Δ*mexEF*, and PA14Δ*mexXY*) and introduced a gentamicin acetyltransferase on the cloning vector pSEVA621 (35) into the respective mutants, the PA14 wild type (WT), as well as into PA14Δ*mexZ*. The introduction of the AME into all strains conferred high gentamicin resistance levels (Fig. 6). The introduction of the AME into Δ*mexCD* or Δ*mexEF* strains did not lead to resistance levels that were different to those of PA14 AME^+^, indicating that these pumps do not play the same important role for the full AME-mediated resistance phenotype as MexXY. However, when the AME was introduced into the Δ*mexAB* mutant, this mutant showed the same phenotype as the Δ*mexXY* AME^+^ strain (Fig. 6), indicating that the MexAB-OprM efflux pump is important for the full resistance potential of the AME. However, this phenotype was lost in the Δ*mexAB* mutant when complemented with *oprM* on the pSEVA634 expression vector (Fig. S4). This suggests that the lack of OprM in the Δ*mexAB* AME^+^ strain, rather than the activity of the MexAB-OprM efflux pump, is responsible for the phenotype.

**FIG 7:**
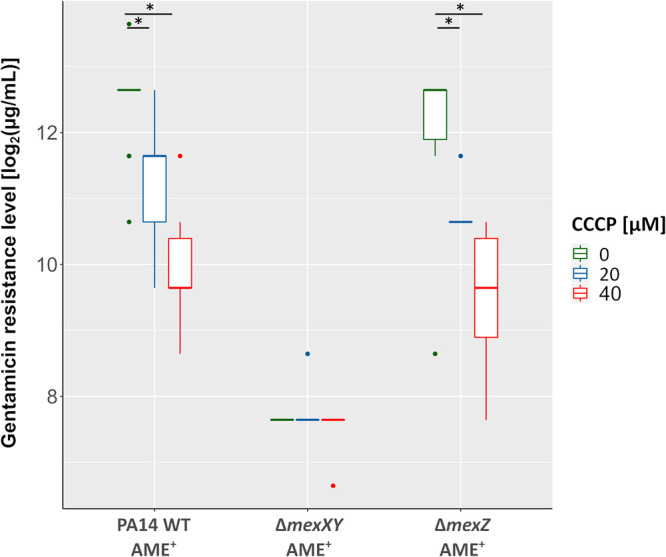
The protonophore CCCP reduces the gentamicin resistance levels of strains with active MexXY-OprM efflux pumps. Gentamicin resistance levels of P. aeruginosa PA14 and mutants thereof. DMSO-dissolved CCCP was added in concentrations of 40 μM, 20 μM, and 0 μM. Each box includes 50% of the data with of least 6 biological replicates. *, *P* < 0.05 (Wilcoxon rank sum test).

We then added the protonophore carbonylcyanid-*m*-chlorphenylhydrazone (CCCP), which has been demonstrated to inhibit efflux pumps (36) to PA14 WT AME^+^, PA14Δ*mexXY* AME^+^, as well as PA14Δ*mexZ* AME^+^, and again recorded the resistance profiles. In agreement with a functional contribution of the efflux pump activity to the high AME-mediated resistance levels, we observed a dose-dependent reduction of gentamicin resistance levels in the PA14 WT AME^+^ and the Δ*mexZ* AME^+^ strains, but not in the Δ*mexXY* AME^+^ strain (Fig. 7). It is important to note that CCCP can also decrease the uptake of aminoglycosides via the disruption of the proton motive force (37–39). However, we did not detect an increase in overall gentamicin resistance upon CCCP addition, indicating that the experimental conditions used in this study are not interfering with aminoglycoside uptake.

In conclusion, our results show that a functional MexXY-OrpM efflux pump is important for the full exploitation of the resistance potential of the horizontally acquired gentamicin acetyltransferase.

## DISCUSSION

Aminoglycosides remain important in the treatment of P. aeruginosa infections, despite their known toxicity. In CF, for example, tobramycin inhalation is applied as a means to control chronic infection and as a first-line treatment for the eradication of early acquisition of P. aeruginosa. Resistance to aminoglycosides is, however, common (33, 40–42). Resistance typically results from drug inactivation by plasmid- or chromosome-encoded aminoglycoside-modifying enzymes (AMEs) or by mutation-driven resistance mechanisms (20, 26, 34). Nevertheless, resistance because of increased efflux is also commonplace, particularly in isolates from CF patients and intensive care units (19, 43–45). Furthermore, aminoglycoside uptake was shown to be inhibited by the presence of mono- and divalent cations (38). Thus, the environment, e.g., in the CF lung, might additionally impact aminoglycoside resistance levels.

The P. aeruginosa genome encodes several multidrug efflux pumps. MexXY-OprM is the only pump that has been described to export aminoglycosides (3, 46). In addition to aminoglycosides, various substances are known to induce the expression of *mexXY* such as fluoroquinolones or cephalosporins (47). Here, we correlated the expression of *mexY*, the substrate specificity-providing component of the MexXY-OprM efflux pump, in 412 clinical P. aeruginosa isolates from various geographical origins and infection sites with their respective tobramycin MICs. The high number of constitutively *mexY*-overexpressing isolates (53%) in our data set clearly shows the importance of the expression of the MexXY-OprM efflux pump in the clinical context. Furthermore, the high number of isolates in our strain collection that did not acquire an AME provided the unique opportunity to evaluate the contribution of *mexXY* gene expression to tobramycin MIC values across a large number of clinical isolates. Indeed, in agreement with previous studies, increased *mexY* expression positively correlated with a decrease in tobramycin susceptibility (27). This effect, however, was limited, and in general, isolates that overexpressed the pump exhibited MIC values that were only 2-fold higher compared to isolates that did not overexpress *mexXY*. In the majority of cases, this did not result in clinically relevant resistance phenotypes. Instead, the main driver of clinically relevant resistance against tobramycin in our set of clinical isolates was horizontally acquired AMEs. They act by either acetylating, nucleotidylating, or phosphorylating specific residues of the aminoglycosides and thereby decreasing the affinity of the compound toward the ribosome (33, 48).

Importantly, we observed a hitherto undetected high correlation between the presence of an AME and overexpression of *mexY*, indicating a relationship of pump overexpression and the exploitation of the full AME resistance potential. We showed that a laboratory strain that lacked the structural genes *mexX* and *mexY* did not gain the same aminoglycoside resistance level when harboring a gentamicin acetyltransferase compared to the wild type or an *mexXY*-overexpressing strain. In agreement, inhibition of efflux pumps via the protonophore CCCP (36) led to a concentration-dependent decrease in aminoglycoside resistance levels in *mexXY*-expressing strains but had no effect on the strain lacking the efflux pump-encoding genes.

Thus, even though the efflux system, on its own, is not a major resistance determinant, it is obviously important for the development of high-level AME-driven resistance against aminoglycoside antibiotics. These results indicate that MexXY is working synergistically with the AME and could be involved in the export of the AME-modified aminoglycosides that otherwise would accumulate in the cell. Nevertheless, it is also conceivable that not the export of the modified aminoglycoside *per se* but the enhanced activity of the efflux pump is important for the full resistance phenotype. In this context, two things are interesting. First, in P. aeruginosa, low extracellular pH increases resistance toward aminoglycosides (49, 50), and second, RND efflux pumps operate as drug/proton antiporters, and their activity has been shown to lead to intracellular H^+^ accumulation (51). Thus, increased activity of MexXY in the presence of an AME and aminoglycosides might lead to increased intracellular proton levels due to an elevated MexXY-driven efflux activity. The efflux pump-driven increase in the intracellular protons might further contribute to aminoglycoside resistance and possibly also to enhanced fitness under a variety of different stressful conditions.

## MATERIALS AND METHODS

### Bacterial strains and growth conditions.

We analyzed efflux pump gene expression in 412 clinical P. aeruginosa isolates that have been previously collected from different laboratories and clinics and for which transcriptional profiles have been recorded (28). Unless otherwise stated, bacteria were grown in standard rich medium culture conditions (LB medium) at 37°C. All strains used in this study are listed in Table S1 in the supplemental material.

### Generation of deletion mutants.

Markerless deletions of structural efflux pump genes were performed as previously described (52). Primers for the amplification of adjacent regions of the targeted genes from UCBPP-PA14 genomic template DNA are listed in Table S2. Briefly, approximately 500 bp located upstream and downstream of the target regions were amplified and then combined in an overlap extension PCR (to generate PA14Δ*mexXY*, PA14Δ*mexCD*, and PA14Δ*mexEF*) (53) or integrated into the pEX18AP cloning vector in a two-step process (to generate Δ*mexAB*). PA14Δ*mexZ*, PA14Δ*mexR*, and the double mutant PA14Δ*mexZ/*Δ*mexR* have been generated in the frame of a previous study (31).

### Sequence variant calling.

We screened for mutations in *mexZ*, encoding the negative regulator of MexXY, in previously published whole-genome sequencing data (29). Mapping was accomplished using Stampy, and variant calling was performed using SAMtools (version 0.1.19) with P. aeruginosa strain UCBPP-PA14 (see “RNA sequencing”) or PAO1 (NCBI assembly accession no. GCA_000006765.1) as a reference. The strain background was assessed based on the phylogenetic analysis documented in Khaledi et al. (29). PA7-like isolates were excluded from this study.

### Detection of aminoglycoside-modifying enzymes.

The resistance genotyping tool ARIBA (version 2.10.2) (54) using the CARD (55) as resistance factor database (downloaded via the ARIBA command “getref card” on 9 November 2020) was employed to detect aminoglycoside-modifying enzymes from the DNA sequencing reads directly in its default settings. We screened the results for resistance factor descriptions containing “aminoglycosides” and excluded hits for *mexZ* as well as the chromosomally encoded APH(3′)-II phosphotransferase from our analysis.

### MexY protein immunodetection.

Overnight grown cultures of clinical isolates (published in reference 30) or PA14 deletion mutants (ΔmexZ or ΔmexR/ΔmexZ) were used to inoculate 10-ml LB main cultures (1:100). After growth to early stationary phase (OD_600_, 1.8 to 2.1), 1 ml of the culture was harvested by centrifugation (6.000 × *g*, 5 min). The pellet was either stored at −70°C or directly suspended in 100 μl lysis buffer (2% sodium dodecyl sulfate, 20 mM Tris hydrochloride, pH 8.0) and boiled at 95°C for 10 min. Residual cells were removed by centrifugation at 21,000 × *g* for 30 min, and the resulting supernatant was sonicated (3 min, setting of 100% ultrasound power, Elma Trassonic water bath) to shear DNA and reduce viscosity. The protein concentration was measured at an absorbance of 280 nm with a NanoDrop spectrophotometer (Thermo Scientific). In total, 60 μg of whole-cell protein solution was mixed 2:1 with SDS sample buffer and heated at 95°C for 10 min before it was applied to a 7.5% SDS-polyacrylamide gel (SDS-PAGE). After separation, the gel was subsequently blotted to a polyvinylidene difluoride (PVDF) membrane (Immobilon-P, Millipore). MexY protein was detected with purified, polyclonal anti-MexY serum (1:20,000) kindly provided by Katy Jeannot, University of Franche-Comté, Besançon, France, followed by a peroxidase-conjugated secondary antibody (anti-rabbit-PO; Dianova; 1:2,000) and Lumi-Light (chemiluminescent peroxidase substrate; Roche) incubation.

### Determination of gentamicin resistance in PA14 efflux mutants.

In order to compare gentamicin resistance levels among the diverse PA14 efflux pump mutants, bacteria were grown for 24 h in Mueller-Hinton 2 medium (Sigma-Aldrich) containing 2-fold dilutions of gentamicin (Roth; concentration range, 50 μg/ml to 12,800 μg/ml) in 96-well plates at 37°C and at constant shaking (180 rpm). Each well was inoculated with an OD_600_ of 0.01. The lowest concentration of gentamicin, which prevented growth of the bacteria to an OD_600_ of 0.1, was recorded.

To inhibit efflux pump activity 40 μM, 20 μM, or 0 μM (control) carbonyl cyanide *m*-chlorophenyl hydrazine (CCCP; Sigma-Aldrich) dissolved in dimethyl sulfoxide (DMSO; Sigma-Aldrich) was added to the medium.

### Data availability.

The RNA sequencing (RNA-Seq) data of the clinical isolates are available from NCBI’s Gene Expression Omnibus (GEO; accession no. GSE122938). The DNA sequencing (DNA-Seq) data of the clinical isolates are available from NCBI's Sequence Read Archive (SRA; accession no. PRJNA526797).

## Supplementary Material

Supplemental file 1
