# Supplementary material for: Expression of the MexXY Aminoglycoside Efflux Pump and Presence of an Aminoglycoside-Modifying Enzyme in Clinical Pseudomonas aeruginosa Isolates Are Highly Correlated
Source: Antimicrob Agents Chemother. 2020 Dec 16;65(1):e01166-20. doi: 10.1128/AAC.01166-20 (PMC7927871; doi:10.1128/AAC.01166-20)
Supplement: Supplemental file 1 [file AAC.01166-20-s0001.pdf]

Supplementary Material

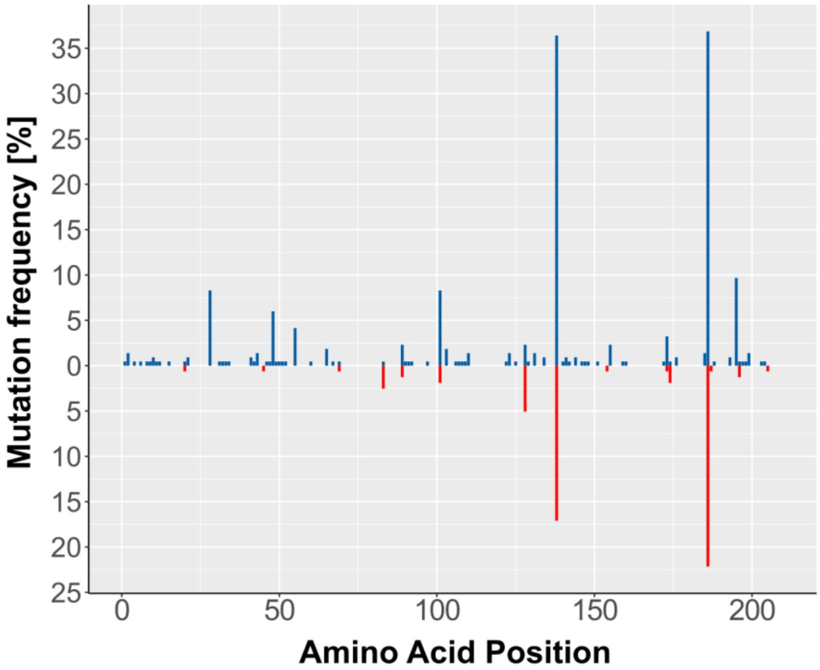

Figure S 1: *mexZ* mutation distribution of *mexY* high and low expressing clinical *P. aeruginosa* isolates. The frequency and localization within the amino acid sequence of non- synonymous mutations of the gene *mexZ* is shownrelative to the number of *mexY* high (blue) and low expressing (red) isolates. Mapping was accomplished using stampy and variant calling was performed using SAMtools (version 0.1.19) with UCBPP-PA14 or PAO1.

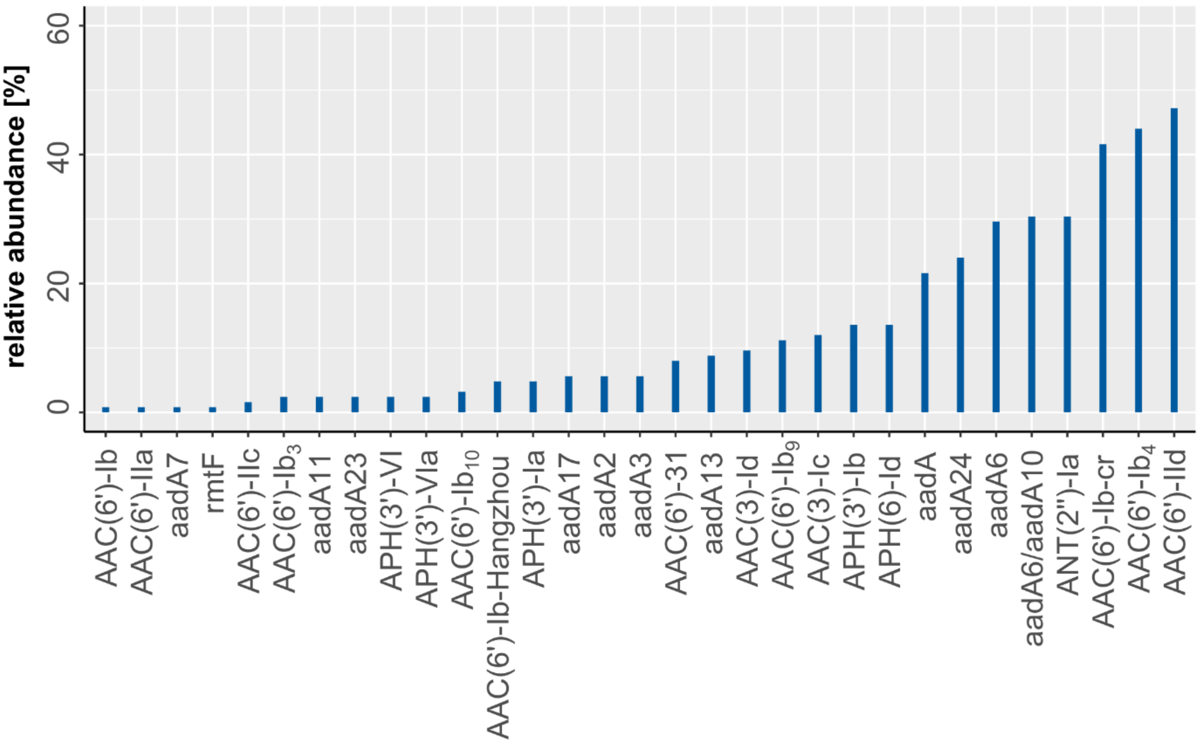

Figure S2: Distribution of detected aminoglycoside modifying enzymes (AME) in our data-set. The resistance genotyping tool ARIBA (version 2.10.2) using the CARD (3) as resistance factor data-base was employed to detect AMEs in the whole genome sequencing data of the analyzed strains. Results were screened for resistance factor descriptions containing "aminoglycosides" and hits for mexZ as well as the chromosomally encoded APH(3')-II phosphotransferase were excluded.

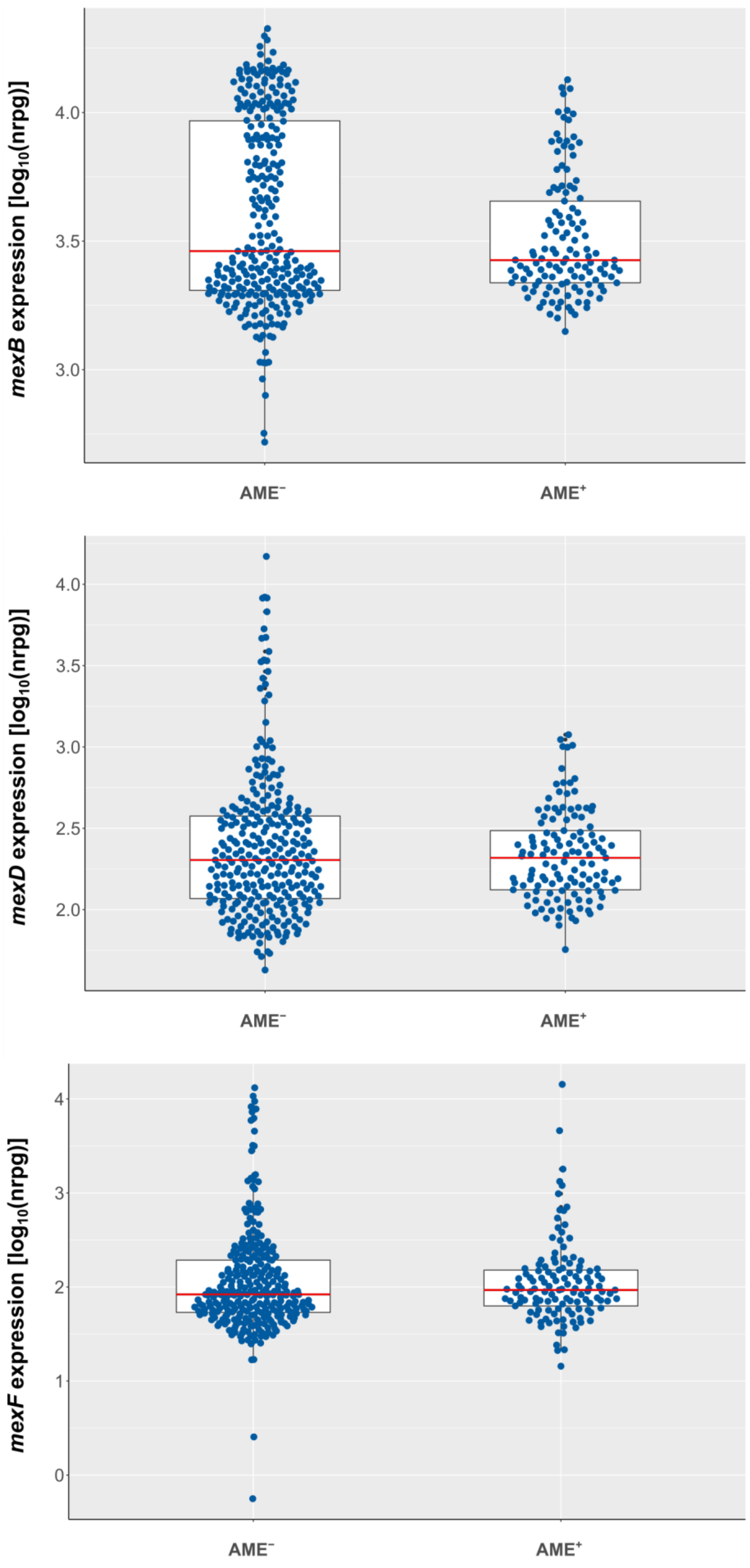
mexB expression [log10(nrpg)]
4.0
3.5
3.0
AME⁻
AME⁺
mexD expression [log10(nrpg)]
4.0
3.5
3.0
2.5
2.0
AME⁻
AME⁺
mexF expression [log10(nrpg)]
4
3
2
1
0
AME⁻
AME⁺

Figure S3: AME possession and efflux pump expression. Normalized expression values (log_{10}(NRPG)) of substrate specificity-providing efflux pump components of clinical *P. aeruginosa* isolates that were identified as AME containing (AME^+) and non AME containing (AME^-). Boxes comprise 50% of the values, red line = median.

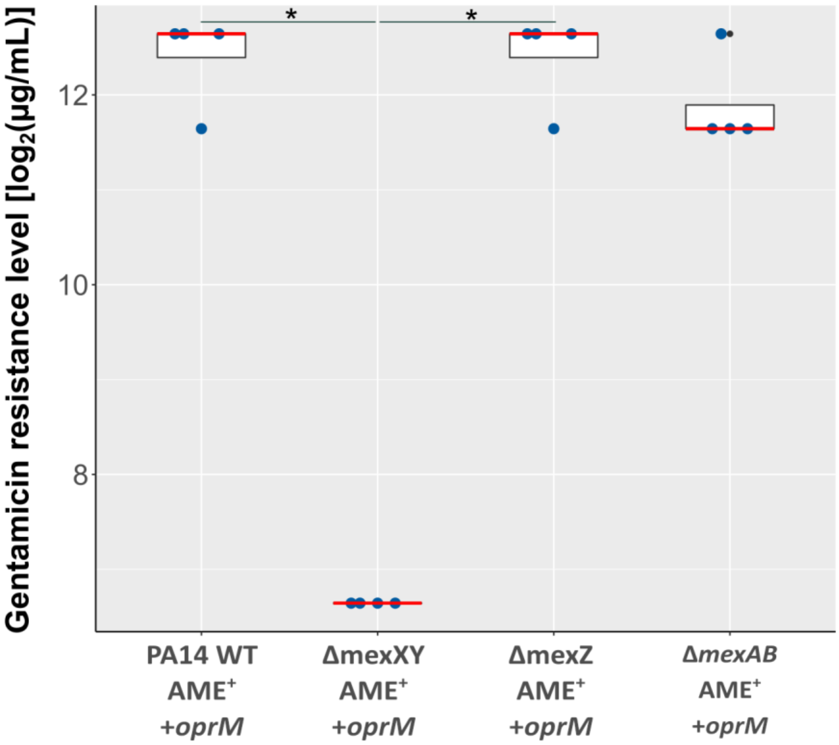

Figure S4: AME induced gentamicin resistance is impacted by the presence of the MexXY-OprM efflux pump. Gentamicin resistance levels of *P. aeruginosa* PA14 and mutants thereof. All strains were transformed with the pSEVA634::oprM vector, harboring a gentamicin acetyl-transferase (AME+) (4). Red line depicts the median resistance level of at least 4 biological replicates; Test for statistical significance: Wilcoxon rank sum test; P-Value < 0.05.

Table S1: Strains used in this study.

Strain	Plasmid	Resistance	Reference
Pseudomonas aeruginosa			
PA14 WT	-	-	(1)
PA14ΔmexAB	-	-	this study
PA14ΔmexXY	-	-	this study
PA14ΔmexCD	-	-	this study
PA14ΔmexEF	-	-	this study
PA14ΔmexZ	-	-	(2)
PA14ΔmexR	-	-	(2)
PA14ΔmexZ/ΔmexR	-	-	(2)
PA14 WT AME^+	pSEVA621	Gm^R	this study
PA14Δ*mexXY*AME^+	pSEVA621	Gm^R	this study
PA14 ΔmexZ AME^+	pSEVA621	Gm^R	this study
PA14 ΔmexCD AME^+	pSEVA621	Gm^R	this study
PA14 ΔmexEF AME^+	pSEVA621	Gm^R	this study
PA14 WT AME^+/+OprM	pSEVA634::oprM	Gm^R	this study
PA14Δ*mexXY* AME+/+OprM	pSEVA634::oprM	Gm^R	this study
PA14 ΔmexZ AME+/+OprM	pSEVA634::oprM	Gm^R	this study
PA14 ΔmexAB AME+/+OprM	pSEVA634::oprM	Gm^R	this study

Gm^R = Gentamicin resistance

Table S2: Primers used in this study.

Target Region	Primer	Sequence	Goal
mexAB	mexAB_FW3-HindIII	GATAAGCTTCAATACATGGACG	knockout of target region
mexAB	mexAB_RW3-BAmHI	TTAGGATCCAAAGGGCCGAAATCG	knockout of target region
mexAB	mexAB_RW2-ECoR	AGCGAATTCTGATGTCCTTGGTCAGTT	knockout of target region
mexAB	mexAB_FW2-BAmHI	TTCGGATCCAGCATATGAAACGGTCCTTC	knockout of target region
mexCD	mexCD_FP1_ECoRI	ATGAATTCTATTCCAGGTAGGACTGCCAGC	knockout of target region
mexCD	mexCD_RP1+20bp	GTTTGCGCATCTCGCTCAGACACACCCGACCGTTGATT	knockout of target region
mexCD	mexCD_FP2+20bp	AATCAACGGTCGGGTGTGTCTGAGCGAGATGCGCAAAC	knockout of target region
mexCD	mexCD_RP2_XbAl	AATTCTAGAACCAGGGCGATCCGTG	knockout of target region
mexEF	mexEF_FP1_ECoRI	AATGAATTCATTCGACGAGGAACTGGAGAAA	knockout of target region
mexEF	mexEF_RP1+20bp	ATCGACTGCGCGTGAATCAGCTTGACTCCGCCAGTCG	knockout of target region
mexEF	mexEF_FP2+20bp	CGACTGGCGGAGTCAAGCTGATTCACGCGCAGTCGAT	knockout of target region
mexEF	mexEF_RP2_XbAl	AATTCTAGAATCAGGCTGACCTGCAGTTG	knockout of target region
mexXY	mexXY_FP1_XbAI	ATCATCTAGATAGCTCGTTCTCGTCGCTGC	knockout of target region
mexXY	mexXY_RP1+20bp	GTTTCGCTAGGGGCATCAGGTGGGTGTCCCTCGATTCGT	knockout of target region
mexXY	mexXY_FP2+20bp	ACGAATCGAGGGACACCCACCTGATGCCCCTAGCGAAAC	knockout of target region
mexXY	mexXY_RP2_HindIII	ACATAAGCTTAGCCTCTACTACGACAAGGCCAG	knockout of target region
oprM	oprM_RBS_EcoRI_L	ACCGAATTCTTTGTTTAACTTTAAGAAGGAGATATACCATGAAACGGTCCTTCCTTTC	amplification of target region
oprM	oprM_clon_HindIII	ACCAAGCTTTCAGGCCTGCGGATCTTCCT	amplification of target region

References

1. Liberati NT, Urbach JM, Miyata S, Lee DG, Drenkard E, Wu G, Villanueva J, Wei T, Ausubel FM. 2006. An ordered, nonredundant library of *Pseudomonas aeruginosa* strain PA14 transposon insertion mutants. Proc Natl Acad Sci U S A 103:2833–2838.

2. Bruchmann S, Dotsch A, Nouri B, Chaberny IF, Haussler S. 2013. Quantitative Contributions of Target Alteration and Decreased Drug Accumulation to *Pseudomonas aeruginosa* Fluoroquinolone Resistance. Antimicrob Agents Chemother 57:1361–1368.

3. Jia B, Raphenya AR, Alcock B, Waglechner N, Guo P, Tsang KK, Lago BA, Dave BM, Pereira S, Sharma N, Doshi S, Lo R, Williams LE, Frye G, Elsayegh T, Sardar D, Westman EL, Pawlowski AC, Johnson A, Brinkman FSL, Wright GD, Mcarthur AG. 2017. CARD 2017 : expansion and model-centric curation of the comprehensive antibiotic resistance database. Nucleic Acids Res 45:D566–D573.

4. Martínez-García E, Aparicio T, Goñi-Moreno A, Fraile S, De Lorenzo V. 2015. SEVA 2.0: An update of the Standard European Vector Architecture for de-/re-construction of bacterial functionalities. Nucleic Acids Res 43:D1183–D1189.
